# Polo-like kinase 1 as a potential therapeutic target and prognostic factor for various human malignancies: A systematic review and meta-analysis

**DOI:** 10.3389/fonc.2022.917366

**Published:** 2022-11-15

**Authors:** Ming-Wen Wang, Zhong Li, Li-Hong Chen, Ning Wang, Jian-Ming Hu, Jin Du, Li-Juan Pang, Yan Qi

**Affiliations:** ^1^ Department of Pathology, The First Affiliated Hospital to Shihezi University School of Medicine, School of Medicine, Shihezi University, Shihezi, China; ^2^ Department of Pathology, The First Medical Center of People's Liberation Army (PLA) General Hospital, Beijing, China; ^3^ Department of Pathology, Zhanjiang Central Hospital, Guangdong Medical University, Zhanjiang, China

**Keywords:** PLK-1, malignant neoplasm, prognosis, survival, meta-analysis

## Abstract

**Objective:**

The overexpression of polo-like kinase 1 (PLK-1) has been found in a broad spectrum of human tumors, making it an attractive prognostic tumor biomarker. Nowadays, PLK-1 is considered a cancer therapeutic target with clinical therapeutic value. The aim of the present study was to systematically review the prognostic and therapeutic value of PLK-1 in different malignant neoplasms.

**Methods:**

A systematic literature search of the Cochrane Library, PubMed, Web of Science, and China National Knowledge Internet (CNKI) databases was conducted between December 2018 and September 2022. In total, 41 published studies were screened, comprising 5,301 patients. We calculated the pooled odds ratios (ORs) and corresponding 95%CIs for the clinical parameters of patients included in these studies, as well as the pooled hazard ratios (HRs) and corresponding 95% CIs for 5-year overall survival (OS).

**Results:**

Our analysis included 41 eligible studies, representing a total of 5,301 patients. The results showed that overexpression of PLK-1 was significantly associated with poor OS (HR, 1.57; 95% CI, 1.18–2.08) and inferior 5-year disease-free survival/relapse-free survival ((HR, 1.89; 95% CI, 1.47–2.44). The pooled analysis showed that PLK-1 overexpression was significantly associated with lymph node metastasis, histological grade, clinical stages (*p* < 0.001 respectively), and tumor grade (*p* < 0.001). In digestive system neoplasms, PLK-1 overexpression was significantly associated with histopathological classification, primary tumor grade, histological grade, and clinical stages (*p* = 0.002, p = 0.001, *p* < 0.0001, respectively). In breast cancer, PLK-1 was significantly associated with 5-year overall survival, histological grade, and lymph node metastasis (*p* < 0.001, *p* = 0.003, *p* < 0.001, respectively). In the female reproductive system, PLK-1 was significantly associated with clinical stage (*p* = 0.011). In the respiratory system, PLK-1 was significantly associated with clinical stage (*p =* 0.021).

**Conclusion:**

Our analysis indicates that high PLK-1 expression is associated with aggressiveness and poor prognosis in malignant neoplasms. Therefore, PLK-1 may be a clinically valuable target for cancer treatment.

## 1 Introduction

Cancer remains one of the leading causes of death worldwide, largely because of tumor cells’ unlimited replication potential and ability to resist apoptosis and escape immune destruction (1). The International Agency for Research on Cancer estimated an incidence of about 19.3 million new cancer cases and 10.0 million cancer deaths in 2020 alone (2). One of the most serious problems associated with cancer treatment is multidrug resistance (MDR), is a common cause of chemotherapy failure and cancer recurrence with a very low survival rate (3). Improvements in cancer diagnosis and treatment therefore represent one of the greatest challenges facing researchers in the coming decades.

Polo-like kinase (PLK) is a serine/threonine protein kinase that is widely expressed in eukaryotic cells. It includes five family members (PLK-1, PLK-2, PLK-3, PLK-4, and PLK-5), of which PLK-1 has been the most extensively studied. PLK-1 is key for cell division, mitotic progression, and DNA damage repair (4, 5). In addition, recent research has found that PLK-1 is also related to epithelial–mesenchymal transition, cell death, and the immune system (6). This protein kinase is differentially expressed in a variety of human cancers, and an increasing number of studies have shown that PLK-1 overexpression is associated with tumor progression and patient prognosis (7). To date, several inhibitors of PLK-1, such as BI2536 (8), volasertib (9), onvansertib (10), and rigosertib (11), have been widely used in various tumor studies, and two of them (BI2536 and volasertib) have entered phase II clinical trials (8, 9). PLK-1 inhibition, in combination with other targeted drugs such as cisplatin and paclitaxel, has thus become a new strategy for the treatment of malignant tumors. (6) This phenomenon indicates that PLK-1 is widely present in various types of tumors, suggesting that it will be a clinically valuable therapeutic target.

PLK-1 has been postulated as a potential oncogene. Therefore, the aim of this study was to perform a systematic review of the literature and a meta-analysis to evaluate the prognostic value of PLK-1 expression in different cancer types.

## 2 Material and methods

### 2.1 Literature search strategy

A systematic literature search of the Cochrane Library, PubMed, Web of Science, and China National Knowledge Internet (CNKI) databases was conducted between December 2018 and September 2022. The search terms used were as follows: (“PLK1,” “polo like kinase 1,” “plk1,” “PLK-1,” or “plk-1”) and (“cancer” or “tumor” or “neoplasm” or “carcinoma”) with no subheading or language restrictions. The reference lists of related articles were also reviewed.

### 2.2 Inclusion and exclusion criteria

This meta-analysis was performed in accordance with the Preferred Reporting Items for Systematic Reviews and Meta-Analyses (PRISMA) statement (12). The inclusion criteria for each study were as follows (1): detected PLK-1 expression in human tumor tissues, (2) evaluated the correlation between PLK-1 expression and prognosis in any type of human malignant neoplasm, and (3) provided sufficient data to calculate the odds ratios (ORs) and 95% CIs of clinicopathological information or the hazard ratios (HRs) and 95% CIs related to 5-year overall survival. Studies were excluded for the following reasons: (1) lacked the necessary data to obtain information on clinical parameters and 5-year overall survival; (2) contained older and incomplete data from two articles reporting results from the same study; and (3) case reports, review articles, and experiments on animals or cell lines alone.

### 2.3 Data extraction and quality assessment

Data extracted from each study included the first author; publication year; country of origin; cancer type; cutoff value for PLK-1 expression; patients’ age, gender, and population characteristics; tumor size and status of distant metastasis; and method of sample analysis. Clinicopathological information included lymph node metastasis, primary tumor grade, histological grade, and clinical stages. Prognostic information included 5-year overall survival, which was extracted from studies providing univariate and multivariate data analyses. Alternatively, where these data were not provided directly in the study, HRs were extracted from Kaplan-Meier curves using Engauge Digitizer software, version 5.1, as described by Tierney et al. (13) STATA software, version 15.1 (StataCorp, LP, College Station, TX, USA) was used for the pooled HRs or ORs with each corresponding 95% CI. Study quality was evaluated by the Newcastle-Ottawa Quality Assessment Scale (NOS), with score items classified into three major categories: selection, assessment of outcome, and comparability. Scores of 7 and above were considered to correspond to high study quality.

### 2.4 Statistical analysis

In this study, the prognostic values of high PLK-1 expression for multiple human cancer prognoses were estimated by HRs and corresponding 95% CIs. An observed HR > 1 implied that patients with high PLK-1 expression had a worse clinical outcome. Pooled ORs and 95% CIs were used to evaluate the association between PLK-1 expression and clinicopathological features. The heterogeneity of the studies was estimated by chi-squared and *I* ^2^ tests. Where the heterogeneity was not significant (*I* ^2^ < 50% or *p* > 0.1), we used the fixed-effect model. In all other cases, random-effects models were applied. Publication bias was estimated by Begg’s and Egger’s tests (14). A *p*-value of less than 0.05 was considered statistically significant. In each system, no further analysis was performed if the clinicopathological or 5-year overall survival information contained fewer than three references.

## 3 Results

### 3.1 Study characteristics

**Figure 1:**
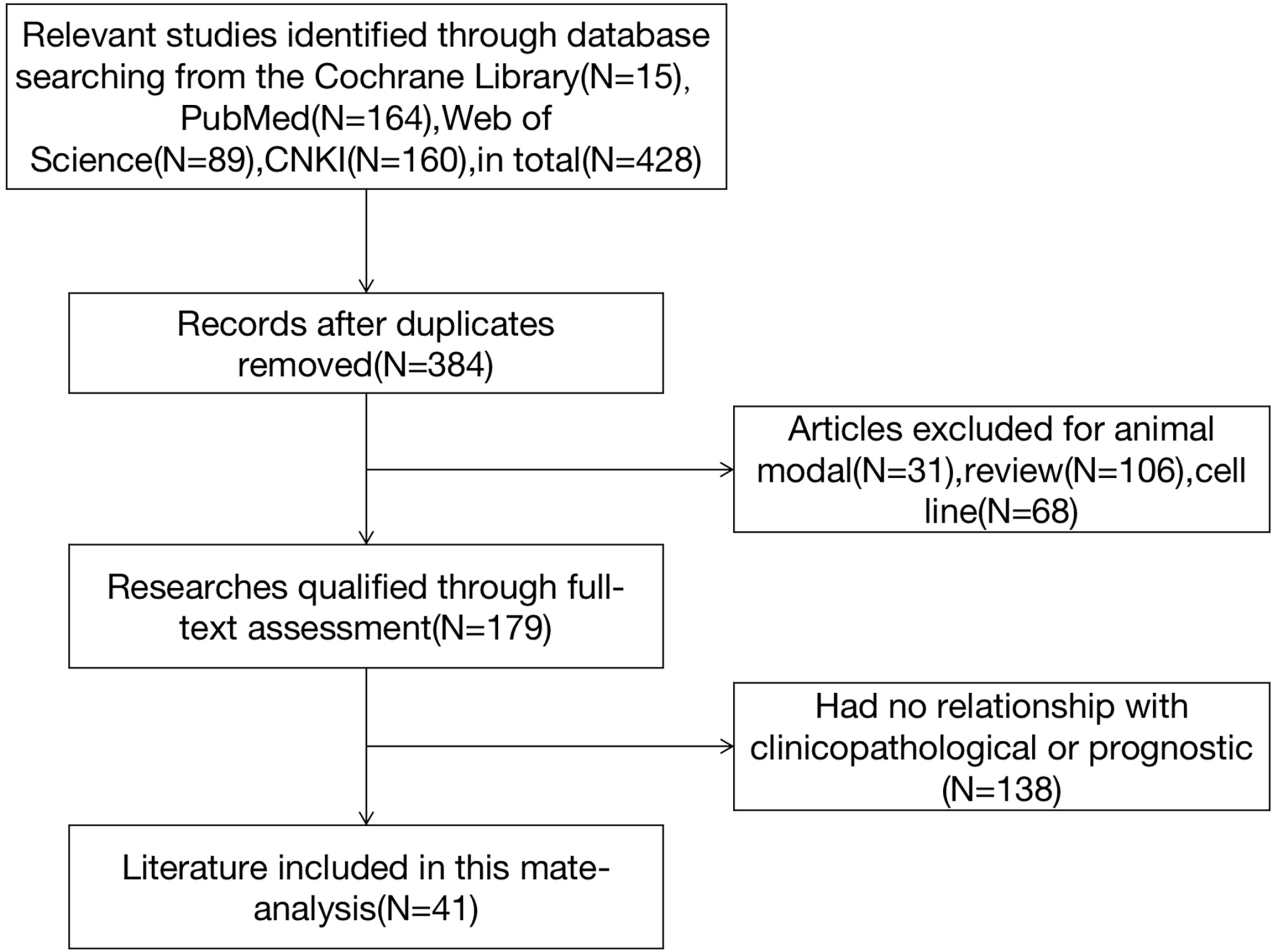
Flow diagram of the study selection process for inclusion in the meta-analysis.

Initially, 428 relevant studies were identified according to the search strategy. From there, 387 articles were excluded for not meeting the inclusion criteria (**Figure 1**). Ultimately, 41 studies were included in this meta-analysis, comprising 29 studies from China, 5 from Germany, 3 from Japan, and 1 each from Spain, England, Australia, and Belgium. In total, 5,301 patients were included in the analysis, representing 3,604 cases from China, 586 from Germany, 445 from Japan, and 666 from the other four countries. The cancer types included hepatocellular carcinoma (HCC), breast cancer (BC), colorectal cancer (CRC), gastric cancer (GC), lung cancer, osteosarcoma, laryngeal neoplasms, ovarian carcinoma, endometrial carcinoma, gallbladder cancer, B-cell lymphoma, malignant glioma, thyroid cancer, pancreatic cancer, and synovial sarcoma (SS). For the detection of PLK-1 expression, quantitative real-time (qRT)-PCR was used in four studies and immunohistochemistry was used in the other 37 studies. The characteristics of all included studies are listed in **Table 1**.

**Table 1:** Characteristics of the studies included in the meta-analysis.

Author	Year	Country	Case	Cancer type	Cut off value	Assay	Score	Data
Kristiansen^27^	2005	Germany	135	BC	6	IHC	7	Both
Kanaji^16^	2006	Japan	160	GC	NA	IHC	8	Both
Sharon^22^	2012	England	215	BC	3	IHC	9	Both
Weichert^26^	2004	Germany	77	Ovarian carcinoma	6	IHC	7	Both
Hajime^20^	2016	Japan	207	GC	6	IHC	7	Both
Cheng^15^	2012	China	68	Malignant glioma	0.88	RT-PCR	8	Both
Takahashi^39^	2002	Japan	78	CRC	NA	IHC	8	Clinicopathological information
Xu^27^	2017	China	266	Lung adenocarcinoma	1	IHC	7	Both
Chen^34^	2009	China	80	GC	1	IHC	7	Clinicopathological information
Chen^36^	2018	China	49	BC	4	IHC	7	Clinicopathological information
Arancha^49^	2016	Spain	75	Rectal cancer	Positive cells ≥10%	IHC	8	Clinicopathological information
Jacob^28^	2005	Germany	86	Pancreatic cancer	6	IHC	7	Both
Fan^44^	2012	China	80	Esophageal neoplasm	Positive cells ≥ 10%	IHC	7	Clinicopathological information
He^32^	2009	China	213	HCC	0.582	RT-PCR	7	Both
Cao^33^	2011	China	70	Laryngeal neoplasms	Positive cells ≥ 6%	IHC	7	Clinicopathological information
Li^24^	2015	China	75	Liver cancer	2	IHC	8	Both
Jolien^40^	2017	Belgium	95	NSCLC	3	IHC	9	Clinicopathological information
He^48^	2012	China	80	B-cell lymphoma	NA	IHC	7	Clinicopathological information
Li^31^	2010	China	167	Endometrial carcinoma	2	IHC	7	Both
Li^51^	2011	China	40	HCC	3	IHC	7	Clinicopathological information
Tut^23^	2015	Australia	281	Rectal cancer	5	IHC	8	Both
Luo^18^	2011	China	84	BC	0.88	RT-PCR	7	Both
Xia^35^	2014	China	80	GC	1	IHC	7	Clinicopathological information
Yang^50^	2018	China	100	HCC	4	IHC	8	Both
Ye^41^	2008	China	63	NSCLC	1	IHC	7	Clinicopathological information
Weichert^30^	2005	Germany	153	Colon cancer	6	IHC	7	Both
Yin^42^	2011	China	87	Esophageal carcinoma	2	IHC	7	Clinicopathological information
Zhang^47^	2014	China	52	Ovarian epithelial cancer	1	IHC	8	Clinicopathological information
Sun^46^	2014	China	67	HCC	NA	RT-PCR	7	Clinicopathological information
Weichert^29^	2006	Germany	135	GC	6	IHC	7	Both
Yun^38^	2017	China	80	BC	4	IHC	7	Clinicopathological information
Guo^45^	2014	China	68	Thyroid cancer	6	IHC	8	Clinicopathological information
Liu^37^	2018	China	803	BC	NA	IHC	8	Clinicopathological information
Li^17^	2017	China	132	Lung carcinoma	6	IHC	7	Both
Wang^25^	2013	China	76	Gallbladder cancer	1	IHC	7	Both
Zhang^43^	2011	China	120	BUC	4	IHC	8	Clinicopathological information
Zhang^44^	2020	China	290	BC	NA	IHC	8	Clinicopathological information
Wei^45^	2021	China	99	GC	2	IHC	8	Clinicopathological information
Yan^46^	2021	China	104	CRC	2	IHC	8	Clinicopathological information
Lu^47^	2022	China	65	Esophageal neoplasm	NA	IHC	7	Clinicopathological information
Li^48^	2020	China	46	SS	2	IHC	7	Clinicopathological information

NA, not available; Both, study contains both clinicopathological and prognostic information; HCC, hepatocellular carcinoma; NSCLC, non-small cell lung cancer; BC, breast carcinoma; CRC, colorectal cancer; GC, gastric cancer; BUC, bladder urothelial carcinoma; SS, synovial sarcoma; IHC, immunohistochemistry.

### 3.2 Meta-analysis of PLK-1 expression and OS

**Figure 2:**
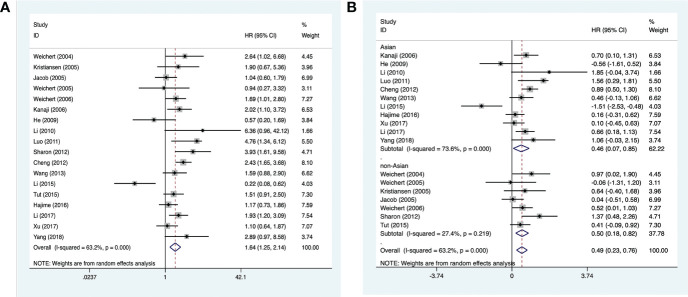
Forest plots of studies evaluating **(A)** the association between PLK-1 expression and 5-year overall survival and **(B)** the 5-year overall survival analysis of PLK-1 expression in Asian and non-Asian patients.

Analysis of PLK-1 and 5-year overall survival in 18 studies (comprising 2,630 cases) (15–32) suggested that high PLK-1 expression (HR, 1.64; 95% CI, 1.25–2.14; *p* < 0.0001; **Figure 2A**) was significantly associated with poor 5-year overall survival in cancer patients. Because the heterogeneity was significant (*I* ^2^ > 50%, *p* < 0.1), we grouped the included literature into “Asia” and “non-Asia” categories to find the source of heterogeneity by subgroup analysis. The results showed that the heterogeneity derived mainly from the Asian literature (**Figure 2B**).

### 3.3 Correlation between PLK-1 and clinicopathological characteristics in cancers

**Figure 3:**
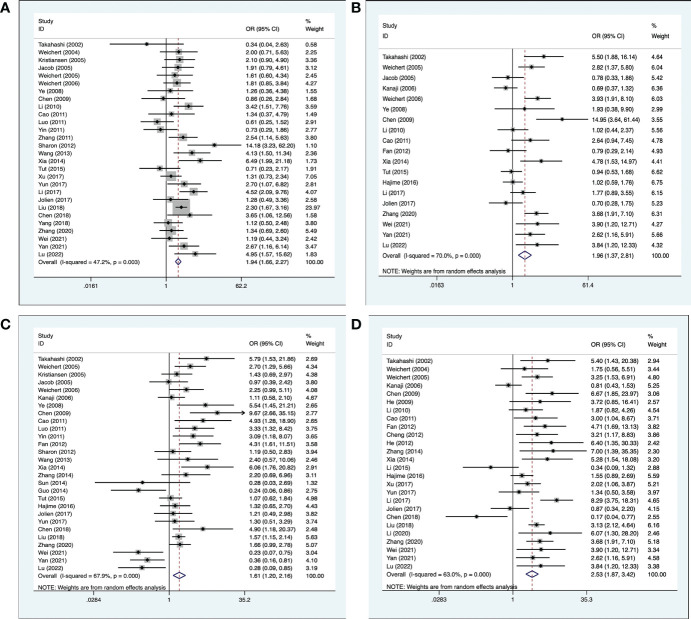
Meta-analysis for the association between PLK-1 expression levels with clinicopathological parameters **(A)** histological grade; **(B)** primary tumor grade; **(C)** lymph node metastasis; **(D)** clinical stage.

Clinicopathological information was extracted from the included 41 studies. Among them, 28 studies (17–19, 21–23, 25–31, 33–47) provided information on the histological grade of the tumor; 19 studies (16, 17, 20, 23, 28–31, 33–35, 39–41, 44–48) provided the primary tumor grade; 28 studies (16, 18, 20, 22, 23, 25, 27–30, 33–42, 44–51) provided information on lymph node metastasis; and 26 studies (15–17, 19, 20, 24, 26, 30–40, 44–48, 51–53) provided information on clinical staging. The results indicated that high expression of PLK-1 was significantly associated with histological grade (OR, 1.94; 95% CI, 1.66–2.27; *p* < 0.001; **Figure 3A**), primary tumor grade (OR, 1.96; 95% CI, 1.37–2.81; *p* < 0.001; **Figure 3B**), lymph node metastasis (OR, 1.61; 95% CI, 1.20–2.16; *p* = 0.001; **Figure 3C**), and clinical stage (OR, 2.53; 95% CI, 1.87–3.42; *p* < 0.001, **Figure 3D**). The details of these results are shown in **Table 2**.

**Table 2:** Main results for the meta-analysis between PLK-1 and clinicopathological features in multiple cancers.

Clinicopathological features	Study (*n*)	Pooled OR (95%CIs)	*z*	*p*-value	Heterogeneity (*I* ^2^)	Heterogeneity (*p*)	Estimated method	Publication bias (*z*)	Publication bias (*p*)
Age (≥ 60/< 60)	7	1.06 (0.76, 1.47)	0.36	0.722	40.9%	0.118	Fixed model	0.30	0.764
Gender (male/female)	24	1.16 (0.97, 1.39)	1.64	0.102	0.0%	0.997	Fixed model	1.07	0.286
Tumor size (≥ 5 cm/<5 cm)	8	1.41 (0.80, 2.49)	1.20	0.232	63.3%	0.008	Random model	0.37	0.711
Distant metastases (yes/no)	9	1.28 (0.53, 3.09)	0.56	0.577	74.3%	0.000	Random model	−0.10	1.000
Histopathological classification (G3/G1–G2)	28	1.94 (1.66, 2.27)	8.38	<0.001	47.2%	0.003	Fixed model	0.06	0.953
Primary tumor grade (T3–T4/T1–T2)	19	1.96 (1.37, 2.81)	3.70	<0.001	70.0%	0.000	Random model	1.61	0.108
Lymph node metastasis (yes/no)	28	1.61 (1.20–2.16)	3.19	0.001	67.9%	0.000	Random model	1.40	0.161
Clinical stages (III–IV/I–II)	26	2.53 (1.87–3.42)	6.06	<0.001	63.0%	0.000	Random model	1.10	0.270

OR, odds ratio; CI, confidence interval.

However, we found no statistically significant relationship between high PLK-1 expression and age (OR, 1.06; 95% CI, 0.76–1.47; *p* = 0.722; **Figure S1A**), gender (OR, 1.16; 95% CI, 0.97–1.39; *p* = 0.102; **Figure S1B**), tumor size (OR, 1.41; 95% CI, 0.80–2.49; *p* = 0.232; **Figure S1C**), or distant metastasis (OR, 1.28; 95% CI, 0.53–3.09; *p* = 0.577; **Figure S1D**).

#### 3.3.1 Digestive system neoplasms

Information on digestive system neoplasms was extracted from 22 studies, comprising 2,517 cases (16–18, 20, 21, 23–25, 28–30, 32, 34, 35, 39, 42, 45–48, 50, 54). Of these, 11 studies (16, 17, 20, 21, 23–25, 28–30, 32) provided information on 5-year overall survival, 14 studies (17, 21, 23, 25, 28–30, 34, 35, 39, 42, 45–47) provided information on the histological grade, 14 studies (16, 17, 20, 23, 28–30, 34, 35, 39, 45–48) provided the primary tumor grade, 16 studies (16, 20, 23, 25, 28–30, 34, 35, 39, 42, 45–48, 55) provided information on lymph node metastasis, and 13 studies (16, 17, 20, 30, 32, 34, 35, 39, 45–48, 56) provided information on clinical staging.

**Figure 4:**
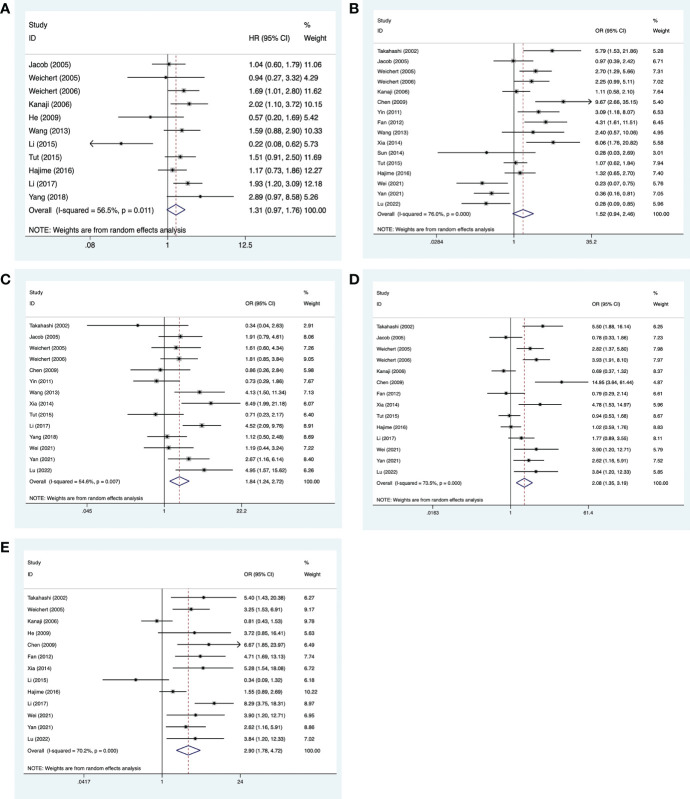
Meta-analysis for the association between PLK-1 expression levels and clinicopathological parameters in cancers of the digestive system. **(A)** 5-year survival; **(B)** lymph node metastasis; **(C)** histological grade; **(D)** primary tumor grade; **(E)** clinical stages.

The analysis showed no significant association between high PLK-1 levels and 5-year overall survival (HR, 1.31; 95% CI, 0.97–1.76; *p* = 0.079; **Figure 4A**) or lymph node metastasis ((OR, 1.52; 95% CI, 0.94–2.46; *p* = 0.089; **Figure 4B**), but the other results indicated that high PLK-1 expression was significantly associated with histological grade (OR, 2.08; 95% CI, 1.35–3.19; *p* = 0.002; **Figure 4C**), primary tumor grade (OR, 2.08; 95% CI, 1.35–3.19; *p* = 0.001; **Figure 4D**), and clinical stage (OR, 2.90; 95% CI, 1.78–4.72; *p* < 0.001; **Figure 4E**). The details of these results are provided in **Table 3**.

**Table 3:** The results for meta analysis between PLK-1 and cancers in different systems.

Cancer type or location	Clinicopathological or prognostic information available	Studies (*n*)	Pooled OR or HR (95% CIs)	*z*	*p*-value	Heterogeneity (I ^2^)	Heterogeneity(*p*)	Estimation method
Digestive system	Five-year overall survival	11	1.31 (0.97–1.76)	1.76	0.079	56.5%	0.011	Random model
	Histopathological classification (G3/G1–G2)	14	2.08 (1.35–3.19)	3.04	0.002	54.6%	0.007	Random model
	Primary tumor grade (T3–T4/T1–T2)	14	2.08 (1.35-3.19)	3.34	0.001	73.5%	< 0.001	Random model
	Lymph node metastasis (yes/no)	16	1.52 (0.94-2.46)	1.70	0.089	76.0%	< 0.001	Random model
	Clinical stages (III–IV/I–II)	13	2.90 (1.78–4.72)	4.28	< 0.001	70.2%	< 0.001	Random model
Breast cancer	5-year overall survival	3	3.60 (2.17–5.97)	4.97	< 0.001	0.6%	0.366	Fixed model
	Histopathological classification (G3/G1–G2)	7	2.12 (1.29-3.50)	2.96	0.003	63.8%	0.011	Random model
	Lymph node metastasis (yes/no)	7	1.64 (1.31–2.05)	4.35	< 0.001	0.0%	0.481	Fixed model
Female reproductive system	Clinical stages (III–IV/I–II)	3	2.22 (1.20–4.13)	2.53	0.011	11.7%	0.322	Fixed model
Respiratory system	Lymph node metastasis (yes/no)	3	2.89 (1.00–8.36)	1.96	0.050	58.5%	0.090	Random model
	Clinical stages (III–IV/I–II)	3	1.75 (1.09–2.82)	2.32	0.021	40.5%	0.186	Fixed model

#### 3.3.2 Breast cancer and the female reproductive system

**Figure 5:**
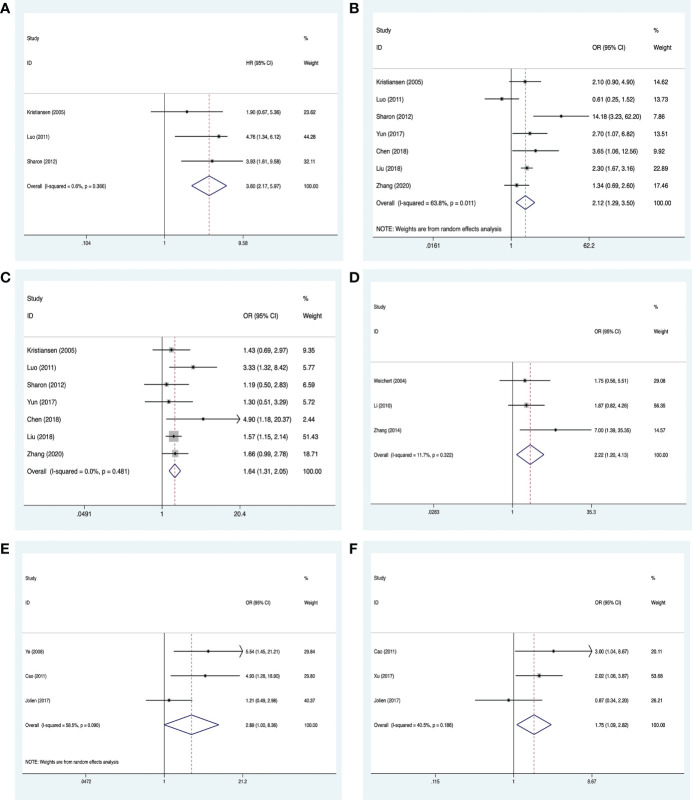
Meta-analysis for the association between PLK-1 expression levels, breast cancer, and cancers of the respiratory system and female reproductive system. **(A)** 5-year overall survival for breast cancer; **(B)** histological grades in breast cancer; **(C)** lymph node metastasis in breast cancer; **(D)** clinical stages in cancers of the female reproductive system; **(E)** lymph node metastasis in cancers of the respiratory system; **(F)** clinical stages in cancers of the respiratory system.

Clinicopathological information on breast cancer was extracted from six studies, comprising 1,656 cases (18, 22, 27, 36–38, 44). An analysis of three of the studies (18, 22, 27) suggested that high PLK-1 expression (HR, 3.60; 95% CI, 2.17–5.97; *p* < 0.001; **Figure 5A**) was significantly associated with poor 5-year overall survival in breast cancer patients. These seven studies also provided information on the histological grade and lymph node metastasis, and an analysis of these factors indicated that high PLK-1 expression was significantly associated with both histological grade (OR, 2.12; 95% CI, 1.29–3.50; *p* = 0.003; **Figure 5B**) and lymph node metastasis (OR, 1.64; 95% CI, 1.31–2.05; *p* < 0.001; **Figure 5C**).

Clinicopathological information on cancers of the female reproductive system was extracted from three studies, comprising 296 cases (26, 31, 51). All three studies provided information on the clinical stage, and our analysis found that high PLK-1 expression was significantly associated with clinical stage (OR, 2.22; 95% CI, 1.20–4.13; *p* = 0.011; **Figure 5D**).

#### 3.3.3 Respiratory system neoplasms

Clinicopathological information on respiratory system neoplasms was extracted from four studies, comprising 494 cases (19, 33, 40, 41). Three of these studies (33, 40, 41) provided information on lymph node metastasis, and three (19, 33, 40) provided information on the clinical stage. The results of our analysis indicated that high PLK-1 expression was not associated with lymph node metastasis (OR, 2.89; 95% CI, 1.00–8.36; *p* = 0.05; **Figure 5E**), but was associated with clinical stage (OR, 1.75; 95% CI, 1.09–2.82; *p* = 0.021; **Figure 5F**). The lack of a significant association with lymph node metastasis may be due to the relatively small number of included studies. The details of these results are shown in **Table 3**.

### 3.4 Publication bias and sensitivity analysis

**Figure 6:**
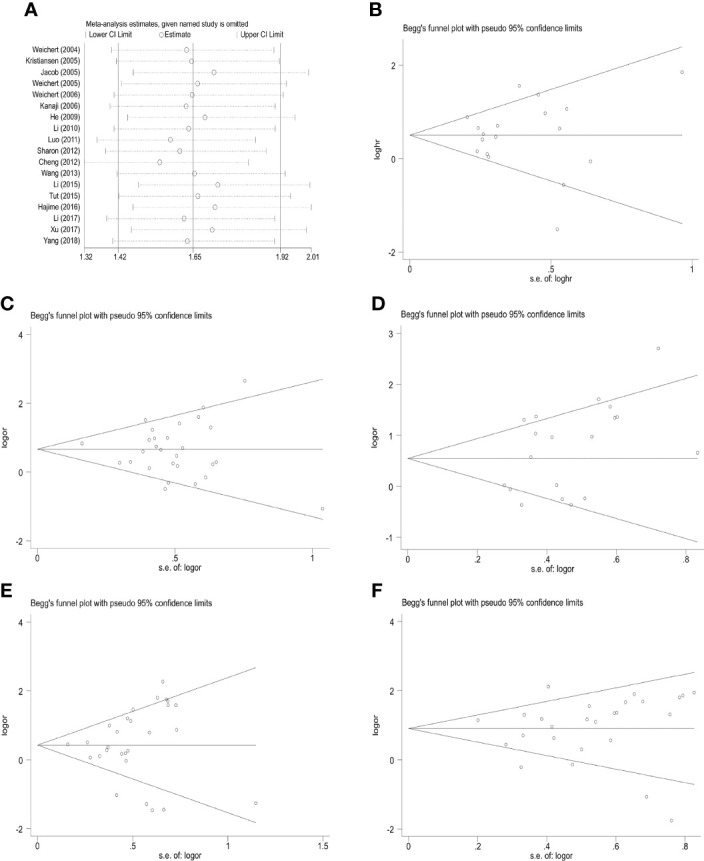
Sensitivity analysis and funnel plot for publication bias testing of the PLK-1 analyses and the clinicopathological parameters of cancer patients. **(A)** sensitivity analysis for 5-year overall survival; **(B)** publication bias plots for analyses of 5-year overall survival; **(C)** histological grade; **(D)** primary tumor grade; **(E)** lymph node metastasis; **(F)** clinical stages.

A sensitivity analysis of the 5-year overall survival studies was conducted by skipping one article per round to evaluate the influence of each data set on high PLK-1 expression and the pooled HRs. The results indicated that there no single study had a disproportionate effect on the combined HRs (**Figure 6**). Additionally, there was no obvious publication bias among all the analyses of PLK-1 expression and 5-year overall survival. The results of Begg’s test for each analysis are presented in **Table 2**.

A sensitivity analysis and an analysis of possible publication bias confirmed the stability of our results. The random-effects model was also used to reduce the impact of heterogeneity on our results.

## 4 Discussion

Prompt, precise, personalized treatment of malignant tumors is critical for improving survival rates and clinical management. PLK-1 has been identified as a treatment target due to its overexpression in various cancers, such as lung, gastric, breast, and colorectal cancer, as well as in other malignancies (52), and its carcinogenic oncogene functions have been confirmed. Some studies have shown that PLK-1 is highly expressed in synovial sarcoma and can promote its occurrence and development (53). On the basis of this extensive literature, PLK-1 has been regarded as a promising prognostic factor in multiple cancers.

We found that there was no statistical significance between PLK-1 expression and 5-year overall survival in digestive system cancers, which was inconsistent with the conclusions of the study by Lin et al. (57), which found that increased expression of PLK-1 was significantly correlated with the survival rate of gastric cancer patients. As a potential prognostic marker of gastric cancer, PLK-1 functions through the MEK-ERK pathway (58).

We also reviewed and compared the results of the meta-analyses of PLK-1 in breast cancer and found that the high PLK-1 expression was significantly different from the tumor size (59). Nevertheless, PLK-1 may become a therapeutic target in the study of triple-negative breast carcinoma (60). The reason for this inconsistent conclusion may be due to the expansion of the research scope, from one cancer to the whole system, leading to differences in the final results. However, in the published meta-analyses on PLK-1, prognoses and pathological stages are affected by the high expression of PLK-1, which is consistent with the results of this study (57, 59).

Several recent investigations have confirmed that PLK-1 plays an important role in cell cycle events, DNA damage repair, epithelial–mesenchymal transition (EMT), and autophagy. During the cell cycle, it is a regulator of mitotic entry and cytokinesis in tumor cells. The significance of PLK-1 in tumor cells is reflected in that its expression often increases when mitosis enters the S phase, reaches its peak in the M phase, and decreases rapidly after mitosis (61). In addition, PLK-1 regulates the initiation and cessation of mitosis by regulating the activities of the cyclinB1/CDK1 complex and APC/C (62, 63). By inhibiting PLK-1 expression, tumor cell mitosis can be arrested in the G2/M phase. Inhibiting PLK-1 in this way also promotes the activation of apoptotic proteins Bax and Bak, causes the inactivation of the anti-apoptotic Bcl-2 protein family, and finally activates caspase-3 and caspase-9 to promote tumor cell apoptosis (64). Recent studies have demonstrated that PLK-1 inhibits DNA damage repair through the P53 signaling pathway and also affects transcriptional processes and apoptotic activity (65). Our investigations have also found that PLK-1 phosphorylates cRAF, which induces the MEK/ERK cascade, eventually activating the ZEB1 and ZEB2 transcription factors, leading to the expression of EMT genes (66). Interestingly, the inhibition of PLK-1 led to autophagy induction through mTORC1 dephosphorylation (67). Due to the effect of PLK-1 on the cell cycle and apoptosis, PLK-1 is highly expressed in tumors and affects the development of tumors and the 5-year overall survival of patients.

The deregulation of the PI3K/Akt pathway, which plays a crucial role in human cancers, has been confirmed. Interestingly, PLK-1 is a downstream gene activated by the PI3K/Akt signal pathway. Mao et al. (68) found that combination therapy, especially therapy targeting the PLK-1/PI3K/AKT pathway, may be a feasible approach for the treatment of pancreatic cancer. Meanwhile, Tan et al. (69) confirmed that PLK-1 is a key member of the pdk1-PLK-1-myc pathway and jointly maintains the growth and differentiation of tumor cells (68). Moreover, PLK-1 can also pass the IKKS of the NF-κB signaling pathway, which is involved in the regulation of normal and tumor cell proliferation. (70) These results indicate that PLK-1 is an important regulator of tumor cell growth and proliferation and can also be used as a target for the treatment of malignant tumors. For this reason, targeting PLK-1 through the development of small molecule inhibitors as anticancer drugs has become an area of intense study.

For example, BI2536, an ATP-competitive inhibitor of PLK-1, has been evaluated for patients in the preclinical setting, with promising results (71). Jeong et al. (72) found that the proliferation and migration ability of breast cancer cells was significantly reduced through the use of BI2536 and by activating the cRaf/ERK signaling pathway, which significantly reduced the cells’ EMT capabilities. Meanwhile, inhibition of PLK-1 expression reduced the forming ability of breast cancer cells and the expression level of tumor stem cell marker proteins (c-myc, Sox2, Oct4, b-catenin, etc.).

By using another PLK-1 inhibitor, BI6727, Dang et al. (73) inhibited PLK-1 expression and caused obvious arrest of the gastric cancer cell cycle in the G2 phase. Moreover, the proliferation and migration of the gastric cancer cells were significantly decreased. BI6727 has therefore been shown to be highly efficacious in inducing tumor regression.

Poloxin and thymoquinone are selective PLK-1 inhibitors targeting the polo-box domain of PLK-1. They can block the correct orientation of PLK-1, thereby preventing cancer cell mitosis (74). Zhao et al. (71) silenced PLK-1 expression by using siRNA, which significantly inhibited the proliferation of esophageal squamous cell carcinoma cells and promoted apoptosis. In addition, clinical trials have shown that using the PLK-1 inhibitors, including BI2536 and volasertib, in combination with decitabine has made some progress against leukemia type 1B (6).

Although this meta-analysis included 41 studies and enrolled 5,301 cancer patients overall, several inherent limitations still exist. First, variability in the detection of PLK-1 expression and subsequent cutoff value selection introduces a potential source of bias. In practice, the lack of a standardized threshold contributes to potential heterogeneity. Second, several studies detected PLK-1 by qRT-PCR, whereas other studies used immunohistochemistry, leading to methodological differences. Third, as some studies did not provide accurate overall survival data, some of the survival data were indirectly extracted from Kaplan-Meier curves *via* software. Accordingly, the corresponding HR and 95% CI may lack credibility.

To summarize, PLK-1 provides significant prognostic value in a number of human malignancies. Overexpression of PLK-1 suggests poor prognosis and aggressiveness. Given the complicated regulatory mechanism between PLK-1 and its target genes, further investigation and additional relevant studies are needed to establish the clinical significance of PLK-1 as aprognostic biomarker and potential therapeutic target.

## Data availability statement

The original contributions presented in the study are included in the article/**Supplementary Material**. Further inquiries can be directed to the corresponding authors.

## Author contributions

M-WW, LZ, and CLH contributed the research concept and design. M-WW, L-JP, and YQ were responsible for developing the methodology and for the writing, review, and revision of the paper. NW, JD, and J-MH provided data acquisition, analysis, and interpretation, as well as statistical analysis. YQ provided technical and material support. All authors read and approved the final paper.

## Funding

This work was supported by grants from the National Natural Science Foundation of China (grant no. 81860471), the international cooperation projects of Shihezi University (grant no. GJHZ201710), the Zhanjiang Science and Technology Development Special Fund Competitive Allocation Project—key projects of disease prevention and control (2021A05145), and the Provincial Science and Technology Special Fund (“College items + task list”) project—the special topic for basic and applied research (2021A05236).

## Conflict of interest

The authors declare that the research was conducted in the absence of any commercial or financial relationships that could be construed as a potential conflict of interest.

## Publisher’s note

All claims expressed in this article are solely those of the authors and do not necessarily represent those of their affiliated organizations, or those of the publisher, the editors and the reviewers. Any product that may be evaluated in this article, or claim that may be made by its manufacturer, is not guaranteed or endorsed by the publisher.

## Supplementary material

Supplementary Figure 1 PLK-1 expression with other information: **(A)** age; **(B)** gender; **(C)** tumor size; **(D)** distant metastases.
