# Supplementary figures and images for: Polo-like kinase 1 as a potential therapeutic target and prognostic factor for various human malignancies: A systematic review and meta-analysis
Source: Front Oncol. 2022 Nov 15;12:917366. doi: 10.3389/fonc.2022.917366 (PMC9705981; doi:10.3389/fonc.2022.917366)

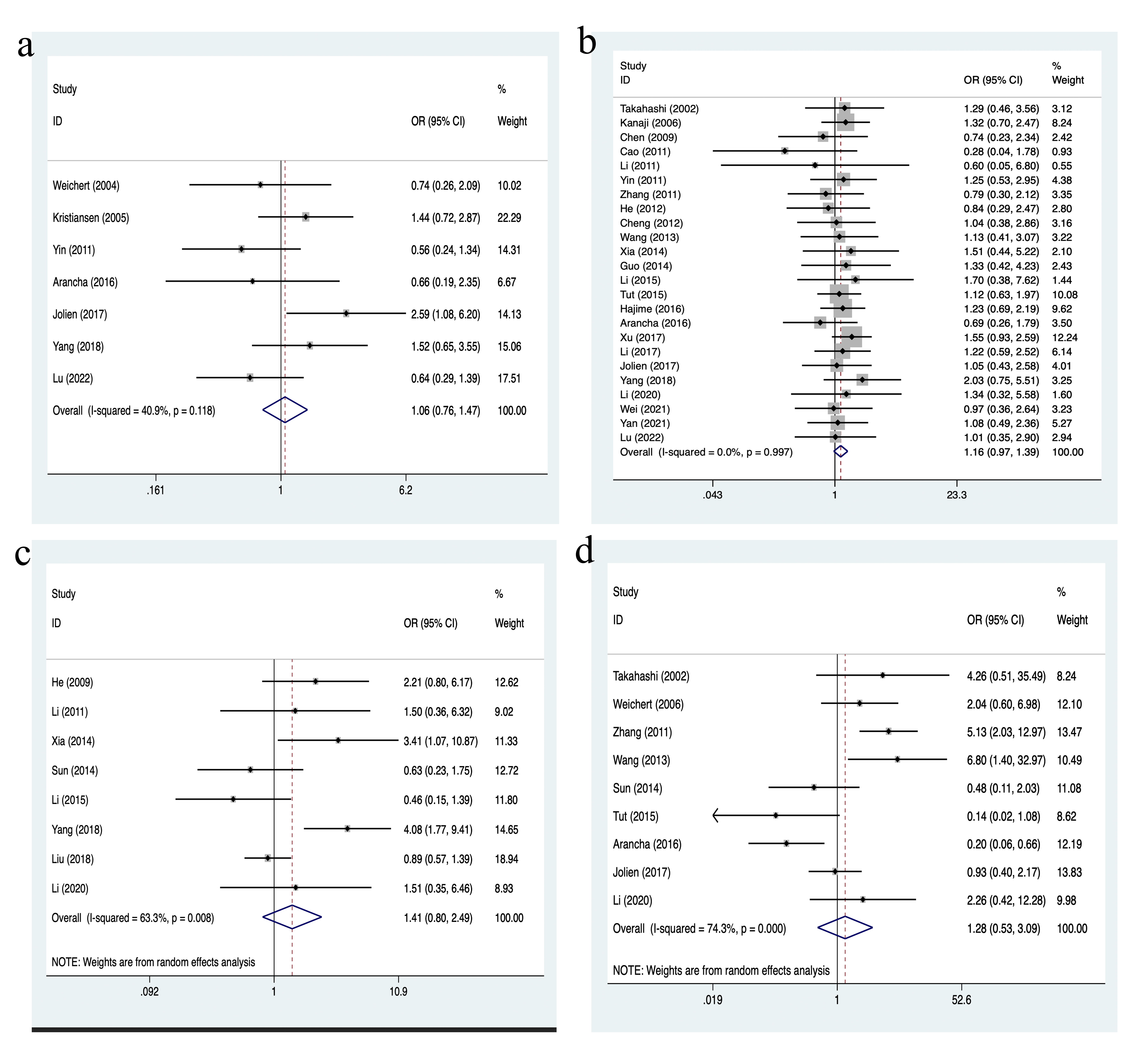

Supplement: Supplementary Figure 1 — PLK-1 expression with other information: (A) age; (B) gender; (C) tumor size; (D) distant metastases. [file Image_1.jpeg]
